# Neutrophil-to-Lymphocyte Ratio Reflects Hepatic and Nutritional Recovery in Hospitalized Patients With Anorexia Nervosa

**DOI:** 10.7759/cureus.104254

**Published:** 2026-02-25

**Authors:** Harue Goto, Kazuhiko Yamamuro, Takahira Yamauchi

**Affiliations:** 1 Department of Psychiatry, Nara Prefecture General Medical Centre, Nara, JPN; 2 Center for Health Control, Nara Medical University Hospital, Kashihara, JPN; 3 Department of Psychiatry, Nara Medical University School of Medicine, Kashihara, JPN

**Keywords:** anorexia nervosa, hepatic function tests, neutrophil-to-lymphocyte ratio, nutritional rehabilitation, refeeding syndrome

## Abstract

Objective: This study aims to investigate changes in the neutrophil-to-lymphocyte ratio (ΔNLR) during inpatient nutritional rehabilitation and to examine its associations with nutritional and hepatic recovery in patients with anorexia nervosa (AN).

Materials and methods: We retrospectively reviewed the medical records of female inpatients aged <20 years admitted for AN at two psychiatric departments in Nara, Japan, between April 2010 and October 2023. Body mass index (BMI) and biochemical parameters were assessed within one week of admission and prior to discharge. NLR was calculated as the ratio of neutrophil to lymphocyte counts. ΔBMI and ΔNLR were defined as the differences between admission and discharge values. Statistical analyses included Wilcoxon signed-rank tests, Spearman’s rank correlation, and multiple linear regression.

Results: BMI increased significantly during hospitalization, as did NLR. At discharge, BMI was negatively correlated with NLR. Lower baseline BMI predicted a greater increase in BMI, whereas higher baseline NLR predicted a greater increase in NLR. ΔNLR were associated with hepatic enzyme dynamics, characterized by increased aspartate aminotransferase (AST) and decreased alanine aminotransferase (ALT), and by the ΔAST/ΔALT ratio, indicating coordinated improvements in hepatic and immune function.

Conclusions: NLR increases in parallel with nutritional and hepatic recovery during inpatient treatment for AN. These findings suggest that NLR may serve as a simple and cost-effective biomarker reflecting systemic physiological restoration beyond weight gain alone.

## Introduction

Anorexia nervosa (AN) is a severe psychiatric illness with the highest mortality among mental disorders [1]. Inpatient nutritional rehabilitation is often required because body weight restoration is essential for medical and psychological recovery. Although a higher body mass index (BMI) at discharge predicts better outcomes [2-3], weight alone does not fully reflect biological restoration [4]. This limitation has prompted growing interest in biological markers that can complement BMI in assessing systemic recovery.

Starvation triggers immunosuppression and low-grade inflammation. Malnourished patients often present with leukopenia or neutropenia from bone marrow suppression, and elevated cytokines such as interleukin-6 and tumor necrosis factor-α have been reported [5]. Psychosocial factors, including early trauma, may further amplify inflammatory responses [6], suggesting that immune activation contributes to the pathophysiology of AN.

Neutrophil-to-lymphocyte ratio (NLR), calculated from routine blood counts, is a simple and reproducible marker of systemic inflammation [7]. Elevated NLR is linked to disease severity and outcomes in psychiatric conditions, including depression and psychosis [8-11]. In AN, however, underweight patients often show a lower NLR that rises with refeeding [12], suggesting that increasing NLR may reflect physiological restoration rather than inflammation. In AN, a low NLR in the underweight state reflects starvation-related suppression of neutrophil production rather than low inflammation. During refeeding, the recovery of hematopoiesis and immune function is accompanied by an increase in NLR, indicating physiological restoration rather than inflammatory activation.

Refeeding in AN also involves substantial hepatic and metabolic adaptations [3,13]. It is well documented that after the initiation of refeeding therapy in severely malnourished individuals, serum aspartate aminotransferase (AST) and alanine aminotransferase (ALT) levels may transiently rise, a phenomenon known as “starvation-induced liver enzyme elevation” [14]. In patients with eating disorders, retrospective studies have reported similar ALT elevations during refeeding, which generally normalize as nutritional status improves [15]. These fluctuations likely reflect transient hepatic stress from metabolic shifts, including the mobilization of fat and glycogen stores and increased oxidative load [13]. While the AST/ALT ratio or the De Ritis ratio is widely used in hepatology to distinguish hepatocellular from mitochondrial injury, its specific behavior during AN refeeding remains less well studied; however, it remains useful in interpreting recovery trajectories, because ALT is predominantly a cytosolic enzyme reflecting hepatocellular injury, whereas AST is present in both the cytosol and mitochondria and is more closely associated with mitochondrial dysfunction and systemic metabolic stress [16]. Hypoalbuminemia frequently occurs in malnutrition but can improve with nutritional rehabilitation, reflecting restoration of hepatic synthetic function [17]. Animal studies indicate that undernutrition reduces albumin (ALB) and total protein (TP) levels, which are recovered after refeeding [18].

Refeeding represents an active therapeutic intervention aimed at restoring systemic physiology in AN, whereas the NLR serves as a readily available biomarker reflecting immune and metabolic recovery. Despite the clinical importance of these changes, the relationship between hepatic and nutritional recovery and inflammatory indices, such as the NLR, remains unclear. Because NLR reflects both immune and metabolic information, it may serve as a holistic marker of recovery. Understanding these relationships could clarify whether the rise in NLR during refeeding reflects inflammatory activation, metabolic adaptation, or a broader physiological restoration.

In this study, we examined longitudinal changes in NLR (ΔNLR) during inpatient treatment for AN and their associations with BMI, hepatic enzyme activity, and nutritional indices during hospitalization. We hypothesized that (1) NLR would increase during refeeding alongside improvements in nutritional status, as suggested by previous studies reporting NLR elevation during nutritional rehabilitation in eating disorders [12,14], and that (2) ΔNLR would be closely linked to improvements in liver function (AST, ALT, and their ratio) and nutritional parameters (TP and ALB), reflecting systemic restoration rather than inflammation alone. The primary objective was to examine longitudinal ΔNLR during inpatient refeeding. Secondary objectives were to explore its associations with hepatic enzyme dynamics and nutritional indices.

## Materials and methods

This retrospective, longitudinal observational study was conducted at the Department of Psychiatry, Nara Medical University School. Hospitalized individuals aged <20 years, admitted between April 1, 2010, and October 31, 2023, were screened. Of the 160 candidates, 87 were included in the final analysis after excluding patients without neutrophil or lymphocyte data. All participants were female. Among them, four were hospitalized three times, and 10 experienced two hospitalizations during the study period; each admission was analyzed independently.

Diagnoses of AN and its subtypes were made at admission by board-certified psychiatrists using the Diagnostic and Statistical Manual of Mental Disorders, Fourth Edition (DSM-IV) [19], Diagnostic and Statistical Manual of Mental Disorders, Fourth Edition, Text Revision (DSM-IV-TR) [20], Diagnostic and Statistical Manual of Mental Disorders, Fifth Edition (DSM-5) [21], or International Classification of Diseases, Tenth Revision (ICD-10) [22] criteria. Of the 87 admissions, 65 (74.7%) were of the restricting type (F50.01), 10 (11.5%) were of the binge eating/purging type (F50.02), and 12 (13.8%) were atypical or other eating disorders (F50.2, F50.8). Hospitalization was determined by attending psychiatrists and pediatricians based on clinical judgment, primarily for medical stabilization, nutritional management, or multidisciplinary inpatient treatment. Participants with an acute infection or inflammatory disease at admission, use of immunosuppressive or anti-inflammatory medications, severe comorbid medical conditions affecting hematological indices, or incomplete clinical data were excluded.

Clinical and laboratory data were retrospectively extracted from electronic medical records. Clinical variables included age, hospitalization duration, and BMI, measured within one week of admission and within two weeks before discharge. BMI was calculated as weight (kg) divided by height squared (m²). Blood samples were collected at the same time points. NLR was calculated as the absolute neutrophil count divided by the absolute lymphocyte count. Serum AST, ALT, TP, and ALB levels were measured using standardized enzymatic assays in the hospital’s central laboratory, with identical analyzers and protocols maintained throughout the study period to ensure consistency.

Behavioral restriction therapy involved monitored meals, activity limitations, and reward-based reinforcement for weight gain in treatment-resistant restrictive-type AN. Nasogastric tube feeding was initiated when oral intake remained insufficient (<1,200 kcal/day) or weight gain plateaued for more than seven consecutive days despite supervised refeeding.

This study was approved by the Ethics Committee of Nara Medical University (approval number: 3996) and conducted in accordance with the Declaration of Helsinki. Given the retrospective design, written informed consent was waived, and an opt-out procedure was implemented on the institutional website.

Statistical analyses

Statistical analyses were performed using SPSS Statistics version 24.0 (IBM Corp. Released 2012. IBM SPSS Statistics for Windows, Version 21.0. Armonk, NY: IBM Corp.). Changes between admission and discharge were examined using the Wilcoxon signed-rank test, and effect sizes (r) were calculated. Associations between BMI and NLR at admission and discharge were assessed using Spearman’s rank correlation coefficients, with differences between correlation coefficients examined using Steiger’s Z-test.

Multiple linear regression analyses identified predictors of changes in BMI (ΔBMI), ΔNLR, and hospitalization duration. Baseline BMI, NLR, nasogastric tube use, and behavioral restriction therapy were the primary independent variables. Admission laboratory variables (AST, ALT, TP, and ALB) were included as covariates in the adjusted model. Additional analyses examined changes in hepatic and nutritional parameters (ΔAST, ΔALT, ΔTP, and ΔALB) and the AST/ALT ratio as predictors of ΔNLR, with ΔBMI and hospitalization duration as covariates. All statistical tests were two-tailed, and a P-value < 0.05 was considered significant.

## Results

Participant characteristics

A total of 87 female patients (mean age = 15.5 ± 2.1 years) were included in this analysis (Table 1).

**Table 1:** Baseline demographic characteristics of the participants Values are presented as mean ± standard deviation. N: number of participants

	Participants (N = 87)
Age, year	15.5 ± 2.1
Gender (male/female)	0/87
Duration of hospitalization, day	81.6 ± 47.7

Changes in clinical parameters between admission and discharge

Wilcoxon signed-rank tests revealed significant improvements in several clinical parameters during hospitalization (Table 2). BMI increased significantly from 13.4 ± 2.7 at admission to 15.5 ± 2.3 at discharge (Z = 7.52, p < 0.001, r = 0.75), indicating a large effect size. Neutrophil counts also increased (21.5 ± 13.3 to 24.7 ± 10.8; p < 0.001, r = 0.44). NLR significantly increased from 1.3 ± 1.1 to 1.8 ± 2.8 (p < 0.01, r = 0.32). Liver enzymes decreased markedly, with AST declining from 79.3 ± 198.7 to 30.9 ± 43.5 (p = 0.01, r = 0.27) and ALT from 56.5 ± 118.6 to 27.2 ± 14.9 (p < 0.01, r = 0.28), both showing medium effect sizes. In contrast, lymphocyte counts, TP, and ALB levels did not change significantly.

**Table 2:** Changes in clinical and laboratory parameters between admission and discharge Values are presented as mean ± standard deviation. Differences between admission and discharge were analyzed using the Wilcoxon signed-rank test. Effect sizes are reported as r = Z / √N and interpreted as small (0.1), medium (0.3), and large (0.5). Statistical significance was defined as a two-tailed p < 0.05. BMI: body mass index, NLR: neutrophil-to-lymphocyte ratio, AST: aspartate aminotransferase, ALT: alanine aminotransferase, TP: total protein, Alb: albumin

	On admission	At discharge	p-value	Effect size (r)
BMI, kg/m²	13.4 ± 2.7	15.5 ± 2.3	<0.001	0.75
Neutrophil count, ×10³/µL	21.5 ± 13.3	24.7 ± 10.8	<0.001	0.44
Lymphocyte count, ×10³/µL	17.7 ± 5.6	17.9 ± 5.8	0.73	0.04
NLR	1.3 ± 1.1	1.8 ± 2.8	<0.01	0.32
AST, U/L	79.3 ± 198.7	30.9 ± 43.5	0.01	0.27
ALT, U/L	56.5 ± 118.6	27.2 ±14.9	<0.01	0.28
TP, g/dL	6.7 ± 0.8	6.7 ± 0.6	0.57	0.07
Alb, g/dL	4.5 ± 0.5	4.3 ± 0.4	0.06	0.20

Relationship between BMI and NLR at admission and discharge

Spearman’s correlation analysis showed no significant association between BMI and NLR at admission (r = -0.09, p = 0.40) (Table 3). However, at discharge, BMI was significantly negatively correlated with NLR (r = -0.32, 95% CI (-0.49, -0.12), p < 0.01). Steiger’s Z-test revealed no significant difference between the correlation coefficients at admission and discharge (Z = 0.68, p = 0.41). ΔBMI and ΔNLR were not significantly correlated (r = 0.05, p = 0.64). Collectively, these results indicate that although NLR was inversely associated with BMI at discharge, this association was not evident at baseline and did not extend to longitudinal changes during hospitalization. This pattern suggests that the increase in NLR during treatment likely reflects physiological recovery processes rather than being directly driven by weight gain itself.

**Table 3:** Correlation analyses between BMI and NLR at admission and discharge Correlation analyses between BMI and NLR at admission and discharge. Spearman’s rank correlation coefficients (ρ) with 95% CI and p-values are shown. Steiger’s Z-test was used to compare the correlation between BMI and NLR at admission with that at discharge. BMI: body mass index, ΔBMI: changes in body mass index, NLR: neutrophil-to-lymphocyte ratio, ΔNLR: changes in neutrophil-to-lymphocyte ratio, CI: confidence interval

Analysis	Spearman ρ	95% CI	p-value
BMI (admission) vs NLR (admission)	-0.09	(-0.29, 0.12)	0.40
BMI (discharge) vs NLR (discharge)	-0.32	(-0.49, -0.12)	<0.01
ΔBMI vs ΔNLR	0.05	(-0.16, 0.25)	0.64

Predictors of ΔBMI during hospitalization

Multiple regression analysis identified baseline BMI as a significant predictor of ΔBMI between admission and discharge (B = -0.31, β = -0.43, p < 0.001; Table 4). Lower admission BMI was associated with greater weight gain during hospitalization. Behavioral restriction therapy was also independently associated with an increased ΔBMI (B = 1.15, β = 0.27, p < 0.01), whereas baseline NLR and nasogastric-tube use were not significant predictors. The crude model explained 36.0% of the variance (adjusted R² = 0.33, p < 0.001). After adjusting for admission laboratory variables (AST, ALT, TP, and ALB), the model remained significant; however, its explanatory power did not improve (adjusted R² = 0.31, p = 0.71). Overall, these results suggest that the improvement in BMI was primarily determined by baseline nutritional status and behavioral intervention, rather than by inflammatory or hepatic parameters.

**Table 4:** Multiple regression analysis of factors associated with ΔBMI between admission and discharge The crude model included BMI on admission, NLR on admission, presence or absence of a nasogastric tube, and behavioral restriction therapy. The adjusted model also included laboratory variables at admission (AST, ALT, TP, and ALB) as covariates. Binary variables were coded as follows: nasogastric tube and behavioral restriction therapy (0 = no (reference), 1 = yes). Model-level p-values represent the overall significance of the regression model based on the F-test. Statistical significance was defined as a two-tailed p < 0.05. BMI: body mass index, ΔBMI: changes in BMI, NLR: neutrophil-to-lymphocyte ratio, SE: standard error, CI: confidence interval

Dependent variables and covariates	B	SE	t-value	p-value	95 % CI
Crude model (R^2^= 0.36, adjusted R^2^= 0.33, p < 0.001)					
BMI on admission	-0.37	0.07	-4.51	< 0.001	(-0.49, -0.18)
NLR on admission	0.14	0.16	0.90	0.37	(-0.11, 0.60)
Presence or absence of a nasogastric tube	0.04	0.39	0.10	0.92	(-0.78, 0.81)
Presence or absence of behavioral restriction therapy	1.15	0.40	2.88	< 0.01	(0.13, 1.81)
Adjusted model (R^2^= 0.38, adjusted R^2^= 0.31, p = 0.71)					
BMI on admission	-0.002	0.002	-1.10	0.28	(-0.005, 0.001)
NLR on admission	0.001	0.003	0.38	0.70	(-0.004, 0.006)
Presence or absence of a nasogastric tube	-0.11	0.51	-0.22	0.83	(-1.13, 0.91)
Presence or absence of behavioral restriction therapy	0.35	0.78	0.45	0.65	(-1.21, 1.91)

Predictors of ΔNLR during hospitalization

Multiple regression analysis revealed that a higher baseline NLR significantly predicted a greater increase in NLR (ΔNLR) (B = 0.59, β = 0.28, p < 0.01; Table 5). In contrast, the BMI at admission and nasogastric tube use were not significant predictors. Behavioral restriction therapy was associated with a negative association with ΔNLR (B = -1.47, β = -0.29, p = 0.01). The crude model explained 17% of the variance (adjusted R² = 0.12, p < 0.01). After inclusion of admission laboratory variables (AST, ALT, TP, and ALB), the model’s explanatory power markedly increased (adjusted R² = 0.62, p < 0.001), indicating that ΔNLR was strongly linked to baseline liver and nutritional parameters. These findings suggest that NLR variations during hospitalization may reflect metabolic and hepatic conditions rather than inflammatory processes.

**Table 5:** Multiple regression analysis of factors associated with ΔNLR between admission and discharge Values are presented as unstandardized regression coefficients (B), SE, t-values, 95% CI, and p-values. The crude model included BMI on admission, NLR on admission, presence or absence of a nasogastric tube, and behavioral restriction therapy. The adjusted model also included laboratory variables at admission (AST, ALT, TP, and ALB) as covariates. Binary variables were coded as follows: nasogastric tube and behavioral restriction therapy (0 = no (reference), 1 = yes). Model-level p-values represent the overall significance of the regression model based on the F-test. Statistical significance was defined as a two-tailed p < 0.05. BMI: body mass index, NLR: neutrophil-to-lymphocyte ratio, SE: standard error, CI: confidence interval

Dependent variables and covariates	B	SE	t-value	p-value	95 % CI
Crude model (R^2^= 0.17, adjusted R^2^= 0.12, p < 0.01)					
BMI on admission	-0.18	0.10	-1.86	0.07	(-0.37, 0.01)
NLR on admission	0.59	0.22	2.73	< 0.01	(0.16, 1.02)
Presence or absence of a nasogastric tube	-0.18	0.54	-0.33	0.74	(-1.23, 0.90)
Presence or absence of behavioral restriction therapy	-1.47	0.56	-2.64	0.01	(-2.52, -0.35)
Adjusted model (R^2^= 0.65, adjusted R^2^= 0.62, p < 0.001)					
BMI on admission	-0.04	0.07	-0.50	0.62	(-0.18, 0.11)
NLR on admission	0.01	0.16	0.05	0.96	(-0.31, 0.33)
Presence or absence of a nasogastric tube	-0.27	0.37	-0.75	0.46	(-1.00, 0.45)
Presence or absence of behavioral restriction therapy	-0.34	0.39	-0.87	0.39	(-1.10, 0.43)

Association between ΔNLR and liver function parameters

Spearman’s correlation analysis revealed significant positive correlations between ΔNLR and both ΔAST (ρ = 0.69, 95% CI (0.54-0.80), p < 0.001) and ΔALT (ρ = 0.54, 95% CI (0.34-0.69), p < 0.001; Table 6), indicating that proportional ΔNLR accompanied improvements in liver enzyme profiles. However, when liver function and nutritional indices were simultaneously entered into a multiple regression model, ΔAST (admission-discharge) and ΔALT showed opposite associations with ΔNLR (Table 7). This discrepancy suggests that while both enzymes correlate positively with NLR in simple correlations, their independent contributions differ when metabolic covariates are controlled, with ΔAST reflecting systemic metabolic recovery and ΔALT reflecting hepatocellular normalization. ΔAST was positively associated with ΔNLR (B = 0.02, β = 1.10, p < 0.001), whereas ΔALT showed an inverse relationship (B = -0.01, β = -0.56, p < 0.001). ΔTP and ΔALB were not significant predictors. The crude model explained 53% of the variance (adjusted R² = 0.51, p < 0.001). After adjusting for ΔBMI and hospitalization duration, the model remained significant (adjusted R² = 0.52, p = 0.17), confirming a robust link between ΔNLR and hepatic enzyme recovery. Collectively, these findings indicate that ΔNLR was closely associated with alterations in liver enzyme patterns during hospitalization, suggesting a potential mechanistic link between immune modulation and hepatic metabolic restoration.

**Table 6:** Spearman’s correlations between ΔNLR and changes in liver enzyme parameters Values are Spearman’s rank correlation coefficients (ρ) with 95% CIs. ΔNLR represents the ΔNLR between admission and discharge, and ΔAST/ΔALT represents the corresponding changes in liver enzyme levels (admission–discharge). Both ΔAST and ΔALT were positively correlated with ΔNLR, suggesting parallel recovery patterns between systemic immune and hepatic functions during nutritional rehabilitation. Statistical significance was defined as a two-tailed p < 0.05. AST: aspartate aminotransferase, ΔAST: changes in aspartate aminotransferase, ALT: alanine aminotransferase, ΔALT: changes in alanine aminotransferase, NLR: neutrophil-to-lymphocyte ratio, ΔNLR: changes in neutrophil-to-lymphocyte ratio, CI: confidence interval

Analysis	Spearman ρ	95% CI	p-value
ΔAST vs ΔNLR	0.69	0.54, 0.80	<0.001
ΔALT vs ΔNLR	0.54	0.34, 0.69	<0.001

**Table 7:** Multiple regression analysis of factors associated with ΔNLR in relation to liver function and nutritional parameters Values are presented as unstandardized regression coefficients (B), SE, t-values, 95% CI, and p-values. The regression model examined factors associated with ΔNLR during hospitalization. The adjusted model included ΔBMI and length of hospital stay as covariates to control for differences in nutritional recovery and treatment duration. All Δ variables were defined as admission minus discharge values to reflect improvement. Model-level p-values represent the overall significance of the regression model based on the F-test. Statistical significance was defined as a two-tailed p < 0.05. Positive Δ values indicate improvement from admission to discharge. SE: standard error, CI: confidence interval, ΔAST: changes in aspartate aminotransferase, ΔALT: changes in alanine aminotransferase, ΔTP: changes in total protein, ΔALB: changes in albumin

Dependent variables and covariates	B	SE	t-value	p-value	95% CI
Crude model (R^2^= 0.53, adjusted R^2^= 0.51, p < 0.001)					
ΔAST (admission–discharge)	0.02	<0.01	7.73	<0.001	(0.01, 0.03)
ΔALT (admission–discharge)	-0.01	<0.01	-3.97	<0.001	(-0.02. -0.01)
ΔTP (admission–discharge)	0.08	0.18	0.47	0.64	(-0.27, 0.43)
ΔALB (admission–discharge)	-0.41	0.40	-1.02	0.31	(-1.20, 0.38)
Adjusted model (R^2^= 0.56, adjusted R^2^= 0.11, p < 0.001)					
ΔAST (admission–discharge)	0.02	0.002	7.66	<0.001	(0.01, 0.02)
ΔALT (admission–discharge)	-0.01	0.003	-4.00	<0.001	(-0.02, -0.01)
ΔTP (admission–discharge)	0.12	0.18	0.67	0.51	(-1.29, 0.32)
ΔALB (admission–discharge)	-0.48	0.40	-1.19	0.24	(-0.09, 0.36)

Association between ΔNLR and the AST/ALT ratio

Using the AST/ALT ratio as a composite index of hepatic function, both the baseline ratio and its improvement (ΔAST/ALT ratio = admission-discharge) were significant predictors of ΔNLR (B = 2.08, β = 0.63, p < 0.001; B = 1.72, β = 0.49, p < 0.001; Table 8). The crude model explained 26% of the variance (adjusted R² = 0.24, p < 0.001). After controlling for ΔBMI and hospitalization duration, the model remained statistically significant (adjusted R² = 0.22, p = 0.83). These findings suggest that ΔNLR is more closely associated with hepatic functional recovery than baseline inflammation, with shifts in NLR and the AST/ALT ratio indicating synchronized improvements in hepatic metabolism during nutritional rehabilitation.

**Table 8:** Multiple regression analysis of factors associated with ΔNLR in relation to the AST/ALT ratio Values are presented as unstandardized regression coefficients (B), SE, t-values, 95% CI, and p-values. The regression model examined factors associated with ΔNLR during hospitalization. Independent variables included the AST/ALT ratio at admission and the ΔAST/ΔALT ratio, defined as the difference between admission and discharge values, reflecting improvement in liver function. The adjusted model additionally included ΔBMI and length of hospital stay as covariates to control for differences in nutritional recovery and treatment duration. Model-level p-values represent the overall significance of the regression model based on the F-test. Statistical significance was defined as a two-tailed p < 0.05. A positive Δ(AST/ALT ratio) value indicates a decrease in the ratio from admission to discharge, reflecting an improvement in liver function. ΔNLR: changes in neutrophil-to-lymphocyte ratio, AST: aspartate aminotransferase, ALT: alanine aminotransferase, BMI: body mass index, SE: standard error, CI: confidence interval

Dependent variables and covariates	B	SE	t-value	p-value	95% CI
Crude model (R^2^= 0.26, adjusted R^2^= 0.24, p < 0.001)					
AST/ALT ratio (on admission)	2.08	0.40	5.23	<0.001	(1.29, 2.87)
AST/LT ratio (admission–discharge)	1.72	0.42	4.10	<0.001	(0.89, 2.55)
Adjusted model (R^2^= 0.26, adjusted R^2^= 0.22, p < 0.001)					
AST/ALT ratio (on admission)	2.04	0.13	4.95	<0.001	(1.22, 2.86)
AST/LT ratio (admission–discharge)	1.66	0.01	3.77	<0.001	(0.79, 2.54)

Predictors of hospitalization duration

Lower baseline BMI and nasogastric tube use were significant predictors of longer hospitalization (B = -3.68, β = -0.25, p = 0.02; B = 22.55, β = 0.26, p = 0.01; Table 9). The crude model explained 17% of the variance (adjusted R² = 0.13, p < 0.01). After adjusting for laboratory covariates (AST, ALT, TP, and ALB), both baseline BMI (B = -4.29, p = 0.01) and nasogastric tube use (B = 18.14, p = 0.04) remained significant (adjusted R² = 0.21, p = 0.03). These findings suggest that more severe nutritional status at admission and the need for enteral feeding are the key determinants of prolonged inpatient treatment. Clinically, these findings underscore the importance of early nutritional intervention in minimizing hospitalization duration and enhancing recovery efficiency.

**Table 9:** Multiple regression analysis of factors associated with the duration of hospitalization Values are presented as unstandardized regression coefficients (B), SE, t-values, 95% CI, and p-values. The crude model included BMI on admission, NLR on admission, presence or absence of a nasogastric tube, and behavioral restriction therapy. The adjusted model also included laboratory admission variables (AST, ALT, TP, and ALB levels) as covariates. Binary variables were coded as follows: nasogastric tube and behavioral restriction therapy (0 = no (reference), 1 = yes). Model-level p-values represent the overall significance of the regression model based on the F-test. Statistical significance was defined as a two-tailed p < 0.05. BMI: body mass index, NLR: neutrophil-to-lymphocyte ratio, SE: standard error, CI: confidence interval, AST: aspartate aminotransferase, ALT: alanine aminotransferase, TP: total protein, ALB: albumin

Dependent variables and covariates	B	SE	t-value	p-value	95% CI
Crude model (R^2^= 0.17, adjusted R^2^= 0.13, p < 0.01)					
BMI on admission	-3.68	1.59	-2.31	0.02	(-6.80, -0.56)
NLR on admission	-4.09	3.56	-1.15	0.25	(-11.07, 2.89)
Presence or absence of a nasogastric tube	22.55	8.88	2.54	0.01	(5.15, 39.95)
Presence or absence of behavioral restriction therapy	3.91	9.17	0.43	0.67	(-14.06, 21.88)
Adjusted model (R^2^= 0.28, adjusted R^2^= 0.21, p = 0.03)					
BMI on admission	-4.29	1.69	-2.54	0.01	(-7.60, -0.98)
NLR on admission	-0.14	3.86	-0.04	0.97	(-7.71, 7.43)
Presence or absence of a nasogastric tube	18.14	8.69	-0.26	0.04	(1.11, 35.17)
Presence or absence of behavioral restriction therapy	-2.34	9.16	-0.04	0.80	(-20.29, 15.61)

## Discussion

In this study, we examined longitudinal ΔNLR during inpatient nutritional rehabilitation in AN. During hospitalization, both BMI and NLR increased, with BMI showing a large effect size (r = 0.75) and NLR a moderate effect size (r = 0.32). Although BMI and NLR were not associated at admission, a significant inverse correlation emerged at discharge. Additionally, ΔNLR were closely linked to normalization of hepatic enzymes and the AST/ALT ratio, suggesting coordinated recovery of immune and metabolic functions during refeeding [23]. The negative correlation between BMI and NLR at discharge indicates that, once nutritional status improves, inter-individual differences in immune recovery may become more apparent and are no longer directly proportional to weight gain. In the context of AN, a low NLR likely reflects starvation-induced suppression of innate immunity and bone marrow activity. In contrast, a rising NLR during refeeding may indicate restoration of neutrophil production and immune competence rather than pathological inflammation.

These findings suggest synchronized recovery of hematologic, hepatic, and nutritional systems during refeeding. Transient elevations in liver enzymes, followed by normalization, have been reported during nutritional rehabilitation [13,14], reflecting metabolic adaptation and immune recovery. Accordingly, rising NLR may indicate coordinated systemic recovery rather than inflammation alone.

Interpretation of findings

The increase in NLR during refeeding is consistent with starvation-related suppression of bone marrow function and innate immunity, resulting in lower baseline neutrophil counts [24]. Nutritional rehabilitation restores hematopoiesis and neutrophil production, leading to an elevated NLR that likely reflects immune recovery rather than pathological inflammation [25]. Although BMI remains the primary indicator of recovery in AN, it does not fully capture biological restoration [5]; in this context, NLR may provide complementary information on systemic recovery during weight restoration [6].

Importantly, the observed increase in NLR was not solely attributable to weight gain but was strongly associated with hepatic parameters, including opposite changes in AST and ALT and an improvement in the AST/ALT ratio. Hepatic enzyme abnormalities are common in malnourished patients with AN and typically normalize with refeeding as hepatocellular integrity and energy metabolism recover [5,26]. The positive association between ΔAST and ΔNLR and the negative association between ΔALT and ΔNLR indicate parallel changes in hepatic metabolic markers and immune-related indices during recovery from starvation.

Transient elevations in AST and ALT during refeeding have been reported in eating disorders and are generally interpreted as short-lived hepatic stress responses during metabolic transition [13]. These changes can be meaningfully interpreted using the AST/ALT ratio, a long-standing index of liver injury patterns [27]. In contrast, serum ALB and TP may recover more slowly or remain within normal ranges despite severe undernutrition; thus, the absence of a significant ΔTP/ΔALB should be interpreted as delayed protein repletion rather than a lack of recovery [28]. Overall, hematological and biochemical abnormalities in AN are largely reversible with appropriate nutritional rehabilitation, supporting the concept of synchronized systemic recovery during refeeding [29].

Clinical implications of hepatic-immune interaction during refeeding

Because ALT predominantly reflects hepatocellular injury, whereas AST is more closely linked to mitochondrial and systemic metabolic activity, changes in the AST/ALT ratio provide insight into the balance between hepatocellular damage and metabolic recovery during refeeding. In the present study, ΔAST was positively associated with ΔNLR, whereas ΔALT was negatively associated with ΔNLR. The parallel ΔNLR and the ΔAST/ΔALT ratio suggest that peripheral immune indices can reflect hepatic recovery during refeeding. Few studies have examined NLR in conjunction with liver function in AN, highlighting the novelty of these findings. The AST/ALT ratio is a well-established marker of hepatic metabolic status [27]. In patients with severe malnutrition, transient ALT elevation during refeeding has been documented [14]. Consistent with these observations, our results indicate that synchronous improvements in NLR and the AST/ALT ratio may reflect restoration of hepatic metabolism and mitochondrial function during nutritional rehabilitation.

The present study revealed opposite associations of liver enzymes with ΔNLR: ΔAST was positively associated, whereas ΔALT was negatively associated. Although both enzymes generally decrease during nutritional recovery, AST and ALT likely reflect distinct physiological processes: AST indexes systemic metabolic and muscular activity, whereas ALT reflects hepatocellular integrity [26]. During refeeding, transient increases in AST may accompany metabolic readjustment, whereas ALT normalization reflects recovery from starvation-induced hepatic injury [5,14]. Accordingly, the positive AST-NLR and negative ALT-NLR associations may indicate coordinated metabolic and immune restoration, a pattern not previously described in AN cohorts [12].

Limitations

This study has several limitations. The female-only, retrospective, single-site design limits generalizability, causal inference, and external validity. NLR is a nonspecific marker influenced by infection, stress, or medication; although active infections were excluded, residual confounding cannot be ruled out. Additionally, direct inflammatory biomarkers such as CRP or IL-6 were not measured, post-discharge outcomes were not assessed, and variability in nutritional rehabilitation protocols may limit exact replication.

## Conclusions

Increases in NLR during refeeding in AN are more closely linked to hepatic and nutritional recovery than to inflammatory activation. The parallel improvement in NLR with hepatic indices suggests coordinated immune and metabolic restoration during nutritional rehabilitation. Because NLR is easily obtained from routine blood tests, it may serve as a practical, integrative biomarker of systemic recovery beyond weight gain alone. Future studies should examine whether longitudinal NLR trajectories predict long-term physiological and clinical outcomes following refeeding.
